# Supplementary material for: The AMIGO1 adhesion protein activates Kv2.1 voltage sensors
Source: Biophys J. 2022 Mar 18;121(8):1395–416. doi: 10.1016/j.bpj.2022.03.020 (PMC9072587; doi:10.1016/j.bpj.2022.03.020)
Supplement: Document S1. Figures S1–S5 and Tables S1–S4 [file mmc1.pdf]

Biophysical Journal, Volume *121*

# Supplemental information

# The AMIGO1 adhesion protein activates Kv2.1 voltage sensors

**Rebecka J. Sepela, Robert G. Stewart, Luis A. Valencia, Parashar Thapa, Zeming Wang, Bruce E. Cohen, and Jon T. Sack**

# Supplemental Material: The AMIGO1 adhesion protein activates Kv2.1 voltage sensors

## Condensed Title: AMIGO1 activates Kv2.1 voltage sensors

Rebecka J. Sepela[1], Robert G. Stewart[1], Luis A. Valencia[3], Parashar Thapa[1], Zeming Wang[3], Bruce E. Cohen[3,4], Jon T. Sack[1,2*]

[1]Department of Physiology and Membrane Biology, University of California, Davis, CA 95616
[2]Department of Anesthesiology and Pain Medicine, University of California, Davis, CA 95616
[3]Molecular Foundry, Lawrence Berkeley National Laboratory, Berkeley, CA 94720
[4]Division of Molecular Biophysics & Integrated Bioimaging, Lawrence Berkeley National Laboratory, Berkeley, CA 94720

*Correspondence to jsack@ucdavis.edu

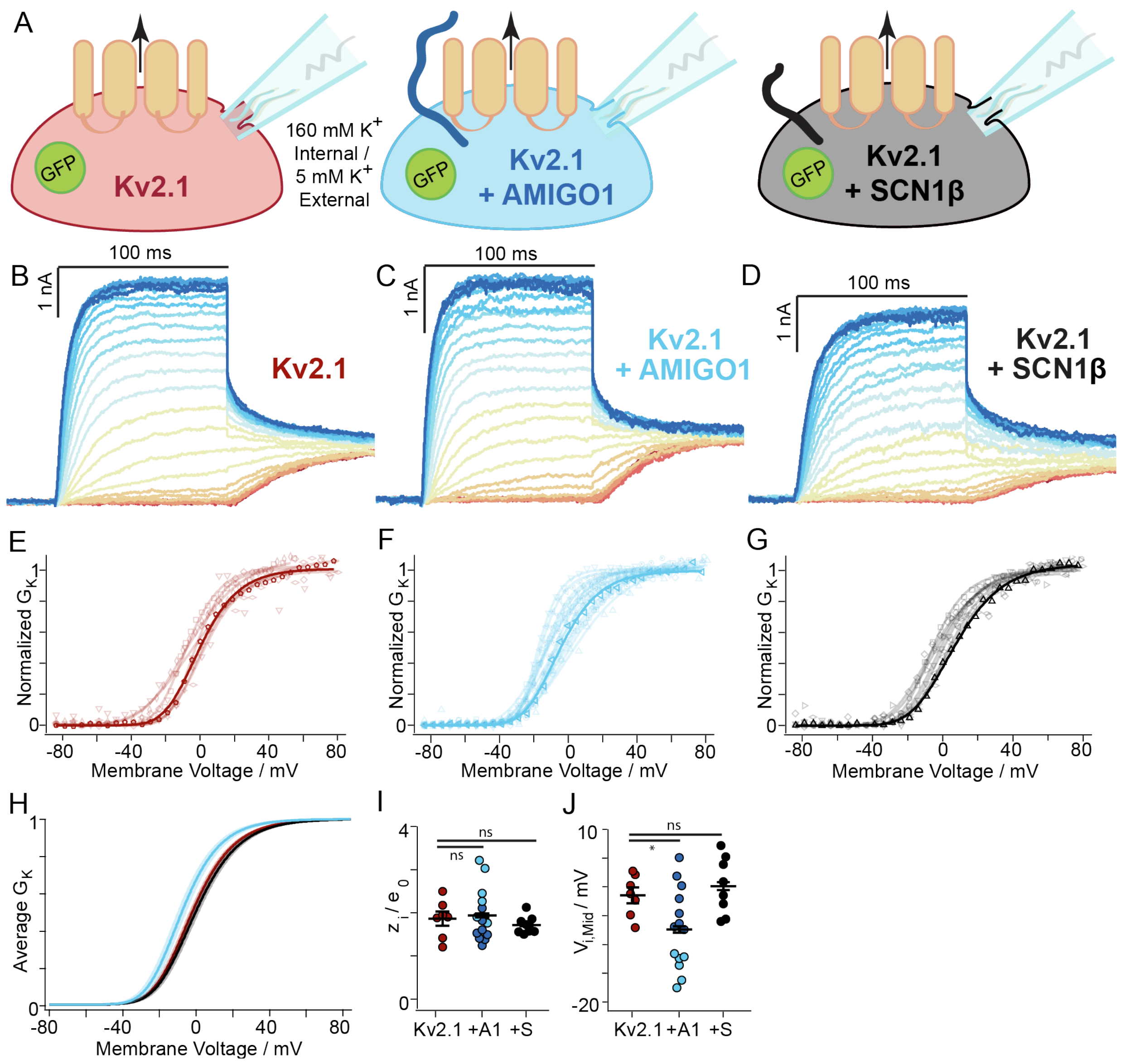


*Supplemental Figure 1. AMIGO1, but not SCN1β, modulates Kv2.1 conductance in HEK293 cells.*

(**A**) Experimental set up: Whole-cell $K^+$ currents from HEK293 cells co-transfected with mKv2.1 and either GFP (red), or AMIGO1–pIRES2–GFP (blue), or SCN1β–pIRES2–GFP (black). (**B**, **C**, **D**) Representative mKv2.1–control (14.8 pF), mKv2.1 + AMIGO1 (9.6 pF), or mKv2.1 + SCN1β (10.0 pF) HEK293 cell. Data points from representative cells are bolded in analysis panels. (**E, F, G**) Normalized *G–V* relationships for mKv2.1–control, mKv2.1 + AMIGO1, or mKv2.1 + SCN1β cells. Symbols correspond to individual cells. Lines are 4$^{th}$ order Boltzmann relationships (Eq. C). (**H**) Reconstructed 4$^{th}$ order Boltzmann fits using the average $V_{i,Mid}$ and $z_i$ (Table 1). Shaded areas represent $V_{i,Mid}$ ± SEM. (**I**) Steepness and (**J**) midpoint of 4$^{th}$ order Boltzmann fits. For the mKv2.1 + AMIGO1 cells, individual $V_{i,Mid}$ and $z_i$ values are displayed in dark or light blue to highlight an increase in variability. Specifically, the standard deviation of $V_{i,Mid}$ increased from ± 3.6 mV in control cells to ± 6.9 mV in mKv2.1 + AMIGO1 cells. We note that the $V_{i,Mid}$ values for mKv2.1 + AMIGO1 cells seemed to partition into two groups: a more negatively shifted group with an average $V_{i,Mid}$ of -13.9 mV (light blue), and a group similar to mKv2.1 alone with an average $V_{i,Mid}$ of -2.5 mV (dark blue). Although all cells analyzed had GFP fluorescence indicating transfection with the AMIGO1–pIRES2–GFP vector, it is possible that some cells were not expressing sufficient AMIGO1 to have a functional effect. Statistics in in Table 1. *: $p = \leq 0.05$, ns: not significant. Bars are mean ± SEM.

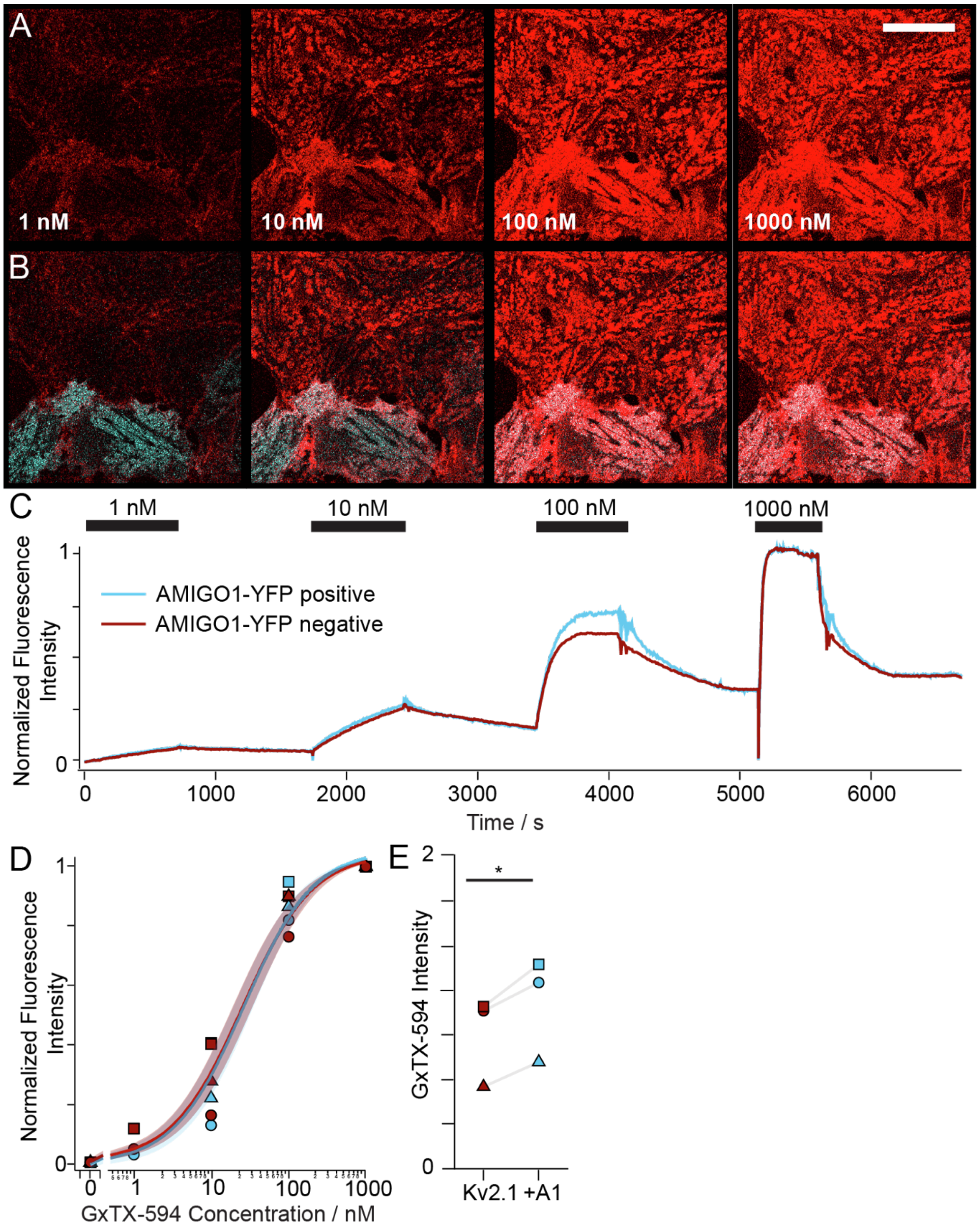


*Supplemental Figure 2. AMIGO1 does not impede GxTX–594 binding to Kv2.1.*

(**A**) Fluorescence from Kv2.1–CHO cells transfected with AMIGO1–YFP, induced for Kv2.1 expression for 48 hours and labeled with indicated concentrations of GxTX–594 (red). Scale bar 20 $\mu$m. (**B**) Overlap (white) between AMIGO1–YFP (cyan) and GxTX–594 fluorescence. (**C**) Mean fluorescence intensities from ROIs encompassing AMIGO1–YFP positive or negative cells from the concentration-response experiment shown in A. (**D**) Normalized fluorescence intensity after 500 s at each concentration as in panel C. Symbol shapes represent data from each of 3 experiments. Curves and shaded regions represent the mean ± SEM of a Langmuir binding isotherm (Eq. L) fit to individual experiments. $K_d$ = 27.5 ± 8.3 nM without and 27.9 ± 7.2 nM with AMIGO1–YFP. $K_d$ likely is overestimated due to incomplete equilibration at 1 and 10 nM. (**E**) Cells expressing AMIGO1–YFP had brighter GxTX–594 fluorescence with 1000 nM GxTX–594. Symbols correspond with D.

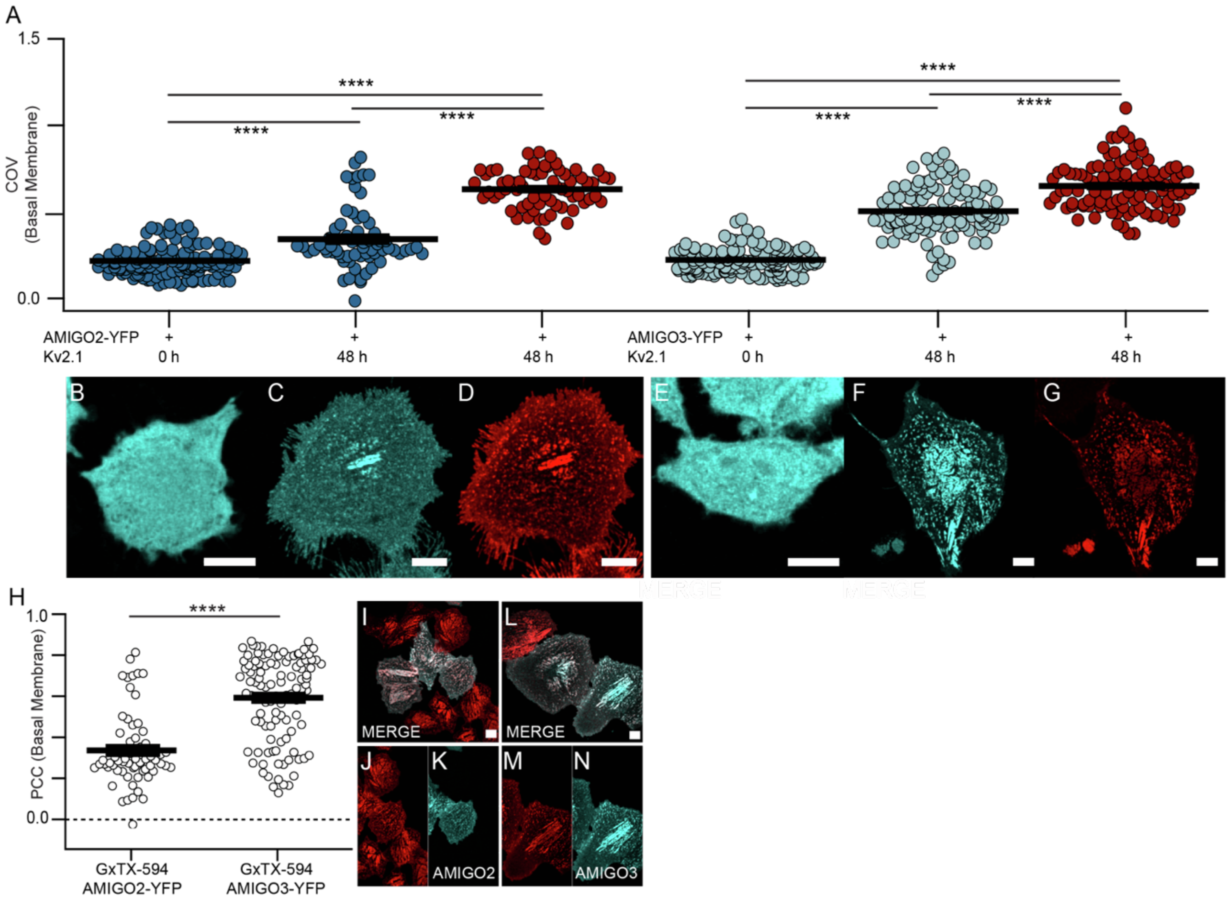


*Supplemental Figure 3. Kv2.1 reorganizes and colocalizes with AMIGO homologs in CHO cells.*
(**A**) Coefficient of variation of fluorescence from AMIGO2–YFP (dark blue circles), AMIGO3–YFP (light blue circles), or GxTX–594 (red circles). COV from confocal images of glass–adhered membranes (exemplar images in **B-G**). AMIGO2–YFP fluorescence from cells (**B**) not induced for Kv2.1 expression ($COV_{A2,0h}$ = 0.2090 ± 0.0062, $n$ = 144), (**C**) induced 48 h for Kv2.1 expression ($COV_{A2,48h}$ = 0.342 ± 0.022, $n$ = 65). (**D**) GxTX–594 labeling of the cells in C ($COV_{A2,48h(GxTX–594)}$ = 0.631 ± 0.013, $n$ = 65 cells). AMIGO3–YFP fluorescence from cells (**E**) not induced for Kv2.1 expression ($COV_{A3,0h}$ = 0.2186 ± 0.0052, $n$ = 160), (**F**) induced 48 h for Kv2.1 expression ($COV_{A3,48h}$ = 0.503 ± 0.014, $n$ = 109). (**G**) GxTX–594 labeling of the cells in panel F ($COV_{A3,48h(GxTX–594)}$ = 0.650 ± 0.013, $n$ = 109 cells). (**H**) Costes thresholded, Pearson's colocalization coefficients from cells induced for Kv2.1 expression 48 h prior to imaging. From left to right: $PCC_{A2,GxTX–594}$ = 0.342 ± 0.022, ≥ 0 ($p <$ 0.0001, one–tailed, t-test), $n$ = 65; $PCC_{A3,GxTX–594}$ = 0.597 ± 0.020, ≥ 0 ($p <$ 0.0001, one–tailed, t-test), $n$ = 108. (**I, J, K**) Exemplar images where merge overlay (white) shows colocalization between GxTX–594 (red) and AMIGO2–YFP (cyan) or (**L, M, N**) AMIGO2–YFP (cyan) Arithmetic means and standard errors are plotted. (**Statistics**) Outliers were removed using ROUT, Q = 1%. An ordinary one-way ANOVA with multiple comparisons was used to evaluate the differences between groups in COV analysis, while a t-test was used to evaluate the PCC data. ****: $p$ = ≤0.0001. Bars are mean ± SEM. All scale bars are 10 $\mu$m.

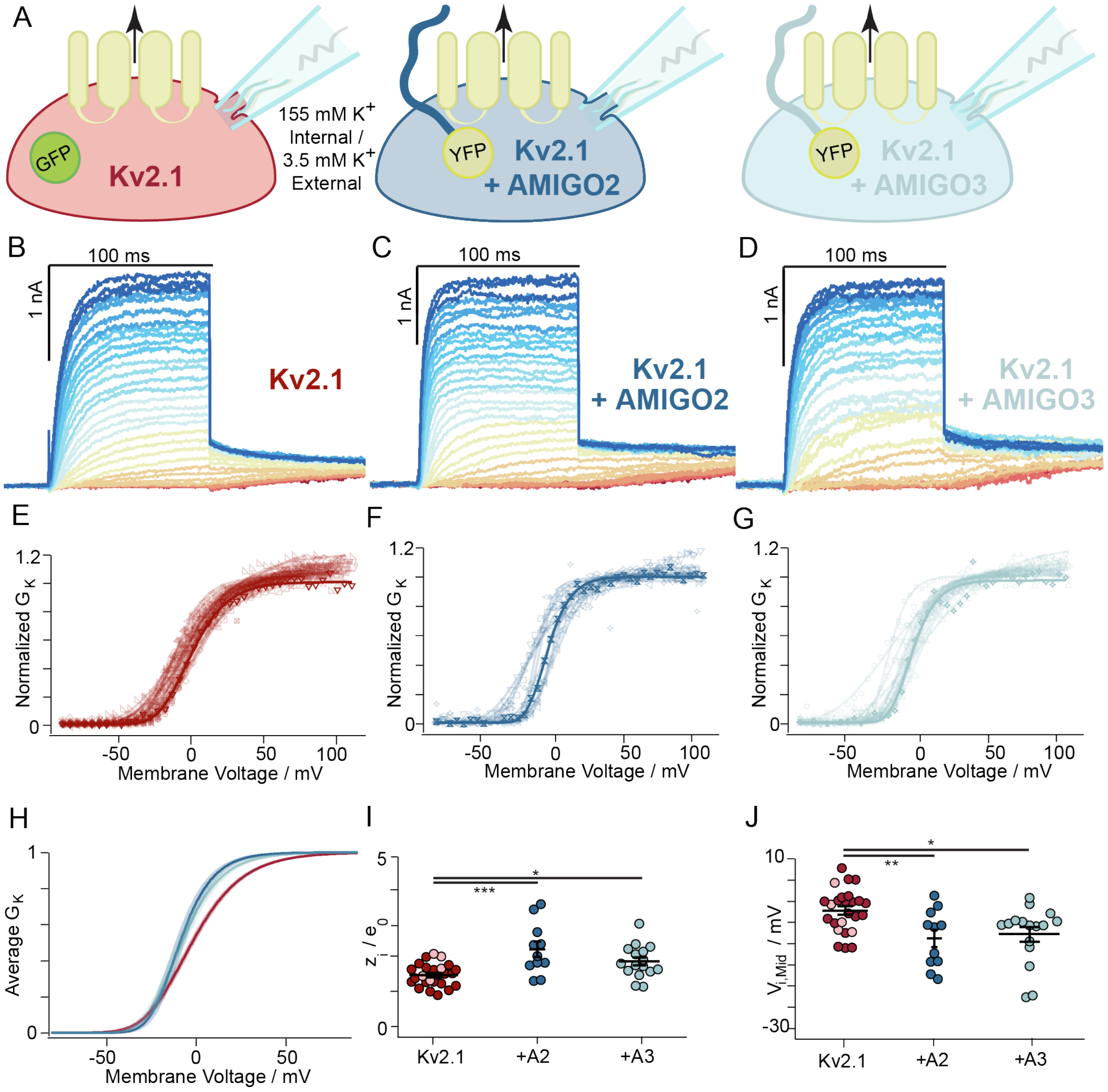


*Supplemental Figure 4. AMIGO2 and AMIGO3 modulate Kv2.1 conductance in CHO cells.*

(**A**) Experimental set up: Whole-cell $K^+$ currents (arrow) from Kv2.1–CHO transfected with GFP (red), rAMIGO2–YFP (dark blue), or rAMIGO3–YFP (light blue). Same voltage protocols and representation as Fig. 3. (**B**, **C, D**) Representative Kv2.1–control (5.1 pF), Kv2.1 + AMIGO2 (6.6 pF) or Kv2.1 + AMIGO3 (2.4 pF) cells. (**E**, **F, G**) Normalized *G-V* relationships. 5 of the Kv2.1–control cells were recorded from side by side with the Kv2.1 + AMIGO2 cells and Kv2.1 + AMIGO3 cells (light red). There was no statistical difference between these 5 cells and the data previously acquired during Kv2.1 + AMIGO1 recordings for Fig. 3 (assessed by t-test), and data was pooled. Solid lines a 4th order Boltzmann fits (Eq. C). (**H**) Reconstructed 4th order Boltzmann fits from average $V_{i,Mid}$ and $z_i$ (Supplemental Table 1). Shading $V_{i,Mid}$ ± SEM. (**I**) Steepness and (**J**) midpoint of fits. Statistics in Table 1. ***: $p = \leq 0.001$, **: $p = \leq 0.01$, *: $p = \leq 0.05$. Bars are mean ± SEM.

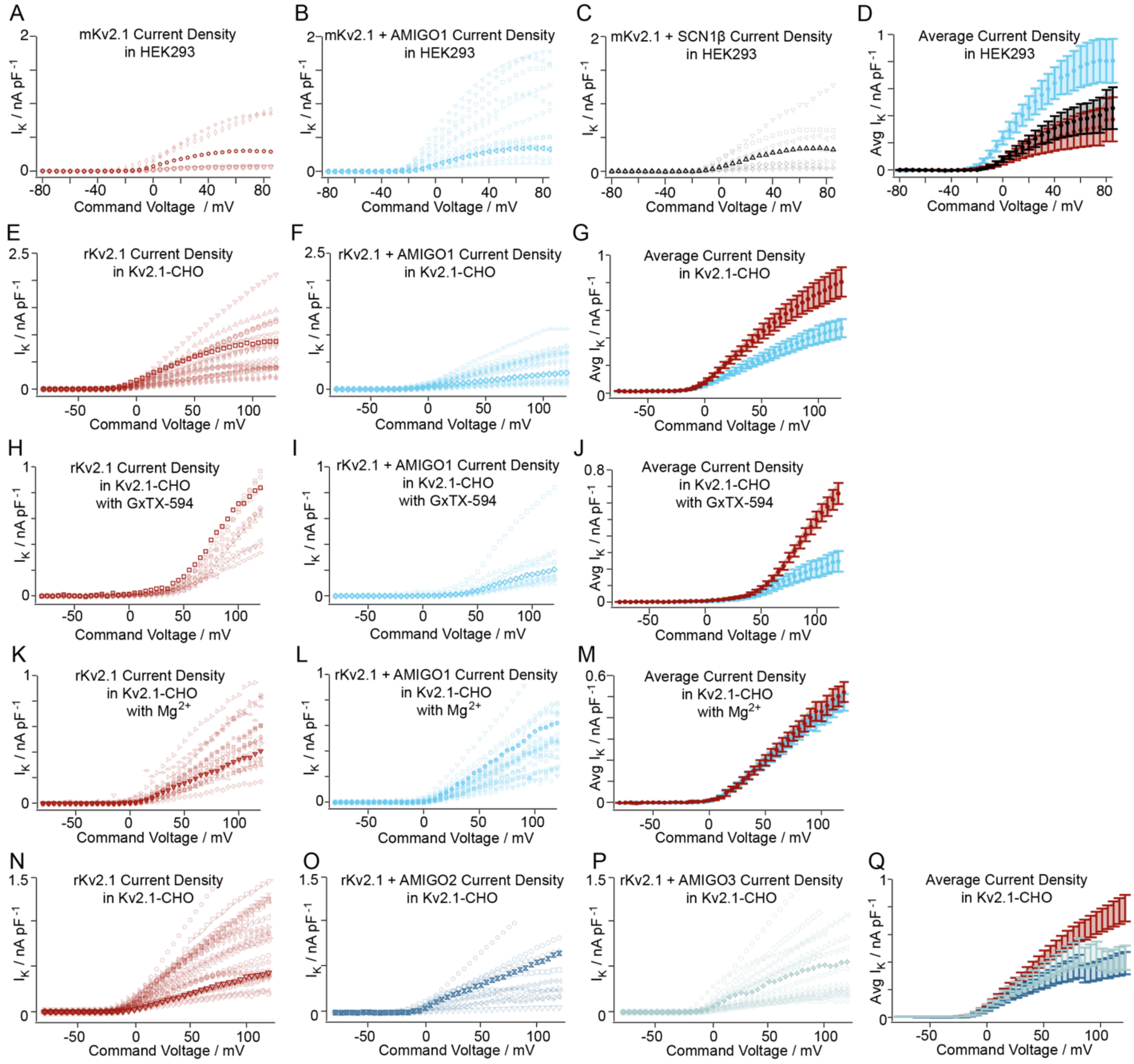


*Supplemental Figure 5. Kv2.1 current density ± AMIGO1 in HEK293 and Kv2.1–CHO cells.*

AMIGO1 has mixed effects on current density in HEK293 and Kv2.1–CHO cells. Outward current densities normalized by cell capacitance were calculated from mean of the last 10 ms of each voltage step and plotted against the command voltage. Symbols represent individual cells. (**A, B, C**) HEK293 cells co-transfected with mKv2.1 + GFP, mKv2.1 + AMIGO1–pIRES2–GFP, or mKv2.1 + SCN1B–pIRES2–GFP. To limit the proportion of currents from endogenous voltage-dependent channels (53, 60), we set a minimum outward current density as an inclusion threshold for analysis (65 pA/pF at +85 mV). Of the cells patched, 7 of 18 mKv2.1–control cells, 14 of 28 mKv2.1 + AMIGO1 cells, and 8 of 27 mKv2.1 + SCN1β cells satisfied this inclusion threshold and displayed currents consistent with a Kv2.1 delayed rectifier conductance ($I_K$). Cells that did not meet the inclusion criteria are not plotted making the full variability of current densities is extreme than depicted here. Bolded symbols are exemplars from Supplemental Fig. 1B, C, or D. (**D**) Averages of A, B, and C. (**E, F**) Kv2.1–CHO ± AMIGO1–YFP. Bolded symbols are exemplars from Fig. 3B or 3C. (**G**) Averages of E and F. (**H, I**) Kv2.1–CHO ± AMIGO1–YFP in 100 nM GxTX–594. Bolded symbols are exemplars from Fig. 5B or 5C. Cell symbols matched between E/H and F/I before and after GxTX–594 addition. (**J**) Averages of H and I. (**K, L**) Kv2.1–CHO ± AMIGO1–YFP in 3.5 mM $K^+$/100 mM $Mg^{2+}$ external. Bolded symbols are exemplars from Fig. 9B or 9C. (**M**) Averages of E and F. Averaged data are means ± SEM.

| | ***G–V* fit parameters** | | | | **$\Delta G_{AMIGOX}$ (kcal/mol)** |
|---|---|---|---|---|---|
| **Kv2.1–CHO cells** | ***$V_{i,1/2}$ (mV)*** | ***$V_{i,Mid}$ (mV)*** | ***$z_i$ ($e_0$)*** | ***n*** | ***(Eq. E)*** |
| **rKv2.1 + GFP** | -32.5 ± 1.5 | -2.0 ± 1.0 $^{A}$ | 1.471 ± 0.067 $^{D}$ | 25 | |
| **rKv2.1+ AMIGO2–YFP** | -29.7 ± 3.4 | -8.7 ± 2.1 $^{B}$ | 2.25 ± 0.23 $^{E}$ | 11 | -0.39 |
| **rKv2.1+ AMIGO3–YFP** | -31.8 ± 2.4 | -7.8 ± 1.7 $^{C}$ | 1.88± 0.12 $^{F}$ | 16 | -0.31 |

*Supplemental Table 1. Fourth order Boltzmann parameters for G–V relationships of AMIGO homologs.*

Average $V_{i,1/2}$, $V_{i,Mid}$, and $z_i$ values were derived from a 4$^{th}$ order Boltzmann fits (Eq. C) of *n* individual cells. All values are given ± SEM. Ordinary one-way ANOVA test with Dunnett's multiple comparisons p-values: AB: 0.0082. AC: 0.010. DE: 0.0002. DF: 0.026. $\Delta G_{AMIGO1}$ from Eq. E, at $V_{i,Mid}$ for Kv2.1 + GFP.

| GxTX(JP) conjugate | AMIGO1–YFP Expression | fitting component | a0 | a1 | a2 | a3 | a4 | a5 | $R^2$ |
|---|---|---|---|---|---|---|---|---|---|
| GxTX Ser13Pra(JP) | - AMIGO | 1 | 0.229 | 670.4 | 47.88 | 11.41 | 1.075 | 2.323 | 0.999 |
| | | 2 | 0.813 | 647.0 | 25.73 | 21.77 | 0.631 | 1.685 | |
| | + AMIGO | 1 | 0.893 | 646.7 | 23.30 | 25.63 | 1.822 | 0.721 | 0.997 |
| | | 2 | 0.006 | -1610 | -15206 | -1877 | 4967 | 461.2 | |
| GxTX Lys27Pra(JP) | - AMIGO | 1 | 0.352 | 594.3 | 12.11 | -11.53 | 0.568 | 5.364 | 0.998 |
| | | 2 | 0.719 | 608.2 | 9.71 | 59.05 | 0.359 | -0.264 | |
| | + AMIGO | 1 | 0.715 | 597.8 | 16.07 | 18.08 | 1.578 | 2.912 | 0.997 |
| | | 2 | 0.632 | 616.3 | 9.05 | 26.28 | -1.657 | 1.488 | |

*Supplemental Table 2. Split Pseudo–Voigt fitting parameters.*

Fluorescence emission spectra split pseudo–Voigt fitting parameters and root-mean squared values.

<table>
<tr><th>Figure</th><th># Transfections</th><th colspan="6">n per transfection</th></tr>
<tr><td>Fig. 3</td><td>7</td><td colspan="3">peGFP: 5, 2, 2, 4, 1, 2, 4</td><td colspan="3">+AMIGO1: 3, 3, 3, 4, 3, 2, 1</td></tr>
<tr><td>Fig. 4</td><td>6</td><td colspan="3">peGFP: 2, 1, 1, 1, 1, 2</td><td colspan="3">+AMIGO1: 1, 2, 1, 3, 0, 0</td></tr>
<tr><td>Fig. 5</td><td>5</td><td colspan="3">peGFP: 4, 2, 2, 3, 2</td><td colspan="3">+AMIGO1: 3, 3, 1, 3, 2</td></tr>
<tr><td>Fig. 6</td><td>6</td><td colspan="3">peGFP: 5, 4, 4, 2, 1, 4</td><td colspan="3">+ AMIGO1: 2, 3, 4, 1, 4, 6</td></tr>
<tr><td>Fig. 7</td><td>2</td><td colspan="3">AMIGO1 (-): 6, 5</td><td colspan="3">AMIGO1 (+): 5, 6</td></tr>
<tr><td>Fig. 9</td><td>4</td><td colspan="3">peGFP: 1, 3, 4, 10</td><td colspan="3">+AMIGO1: 5, 5, 7, 6</td></tr>
<tr><td>Sup. Fig. 1</td><td>4</td><td colspan="2">peGFP: 3, 3, 1, 0</td><td colspan="2">+AMIGO1: 4, 4, 6, 0</td><td colspan="2">+SCNB1: 1, 1, 2, 4</td></tr>
<tr><td>Sup. Fig. 4</td><td>5</td><td colspan="2">peGFP: 5, 0, 0, 0, 0<br>(+peGFP n-values from Fig. 3)</td><td colspan="2">+AMIGO2: 1, 2, 0, 1, 7</td><td colspan="2">+AMIGO3: 1, 7, 5, 0, 3</td></tr>
</table>

*Supplemental Table 3. N-values for electrophysiology experiments.*

| Figure | # Transfections | # *n* values per transfection | | | | | |
|---|---|---|---|---|---|---|---|
| **Fig. 1** | 4 | YFP (0 hr): 28, 48, 0, 58 | YFP (1.5 hr): 25, 55, 42, 95 | YFP (48 hr): 82, 54, 74, 67 | YFP (ChR): 11, 21, 32, 61 | GxTX-594 (48 hr, AMIGO1): 84, 44, 69, 0 | mRuby-ChR (AMIGO1): 20, 16, 32, 60 |
| **Fig. 2** | 4 | AMIGO1-YFP +GxTX-594 (48 hr): 85, 41, 69, 0 | | | AMIGO1-YFP +ChR-mRuby: 18, 22, 28, 61 | | |
| **Fig. 2** | 3 | 0 hr: 41, 35, 25 | | 1.5 hr: 38, 39, 41 | | 48 hr: 28, 17, 56 | |
| **Fig. 8** | 3 | AMIGO1(-) (GxTX Ser27Pra-JP): 20, 12, 8 | | | AMIGO1(+) (GxTX Ser27Pra-JP): 39, 20, 13 | | |
| | 2 | AMIGO1(-) (GxTX Ser13Pra-JP): 15, 55 | | | AMIGO1(+) (GxTX Ser13Pra-JP): 7, 62 | | |
| **Sup. Fig. 2** | 3 | AMIGO1(-): 1, 1, 1 | | | AMIGO1(+): 1, 1, 1 | | |
| **Sup. Fig. 3** | 2 | AMIGO2-YFP (0 hr): 28, 116 | AMIGO2-YFP (48 hr): 59, 6 | GxTX-594 (48 hr, AMIGO2): 59, 6 | AMIGO3-YFP (0 hr): 117, 43 | AMIGO3-YFP (48 hr): 109, 0 | GxTX-594 (48 hr, AMIGO3): 109, 0 |
| **Sup. Fig. 3** | 2 | AMIGO2-YFP +GxTX-594: 64, 1 | | | AMIGO3-YFP +GxTX-594: 108,0 | | |

*Supplemental Table 4. N-values for imaging experiments.*
